# Two‐Staged Bronchoscopic Lung Volume Reduction: A Single US Academic Center Retrospective Analysis Demonstrating Reduction in Pneumothorax

**DOI:** 10.1002/rcr2.70355

**Published:** 2025-09-22

**Authors:** Chih‐Huan Lu, Brooks T. Kuhn, Michael Schivo, Chinh Phan, Ken Y. Yoneda, Jeremy J. Kim

**Affiliations:** ^1^ Division of Pulmonary, Critical Care, & Sleep Medicine University of California Davis School of Medicine Sacramento California USA; ^2^ Veterans Affairs Medical Center Mather California USA

**Keywords:** bronchoscopic lung volume reduction, emphysema, endobronchial valves, interventional pulmonology, pneumothorax

## Abstract

Staged Bronchoscopic lung volume reduction (BLVR) has been proposed to reduce the risk of pneumothorax in patients with emphysema, though evidence to date is limited. We present a retrospective series from a single US academic centre, evaluating pneumothorax and other complications following staged BLVR. Seventeen patients underwent staged BLVR at our centre. Two illustrative cases are presented: (1) a case of pneumothorax post‐staged BLVR managed conservatively without chest tube insertion, and (2) a patient who initially developed pneumothorax after single‐stage BLVR, subsequently completing staged BLVR without complications. Overall, the pneumothorax rate within 45 days was 11.8%, and none of the events required tube drainage or valve removal. This retrospective analysis suggests that staged BLVR may reduce the incidence of pneumothorax. Larger, randomised controlled trials are warranted to confirm these potential benefits.

## Graphical abstract

This retrospective case series suggests a promising reduction in pneumothorax rates and potentially reduced severity following staged BLVR. Larger, randomised controlled trials are warranted to confirm these potential benefits.

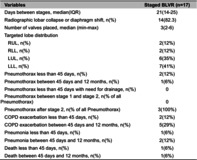

## 1 Introduction

Chronic obstructive lung disease (COPD) affects 10% of the population and is the 4th leading cause of death according to the World Health Organization [1]. Emphysema is one manifestation of COPD that involves lung tissue destruction resulting in air retention and hyperinflation. Bronchoscopic lung volume reduction (BLVR) involves bronchoscopic placement of one‐way endobronchial valves (EBVs), enabling passive deflation of hyperinflated lobes and diaphragm unloading. Successful BLVR improves FEV1 and symptoms, often measured by 6 min walk distance (6MWD), St. George's Respiratory Questionnaire (SGRQ), Modified Medical Research Council (mMRC) dyspnea scale, and/or BODE index [2].

The most common complication of BLVR is pneumothorax, with a 45‐day risk of 26.6% in the pivotal LIBERATE trial [2]. In that trial, 85% of pneumothoraxes occurred within 5 days, and 83% were managed with tube thoracostomy. Similar rates were reported in TRANSFORM (23%) and IMPACT (25.6%) trials [3, 4].

To reduce pneumothorax risk, Egenod et al. proposed a 2‐step BLVR approach in a retrospective multicentre French study [5, 6]. Patients received EBVs in all but the most proximal segments first, followed by valve placement in the remaining segments 4 weeks later. Pneumothorax occurred in 12.5% with no increase in other adverse events. These findings prompted us to assess the feasibility and safety of a staged BLVR protocol at our institution.

## 2 Case Series

### 2.1 Methods

**FIGURE 1:**
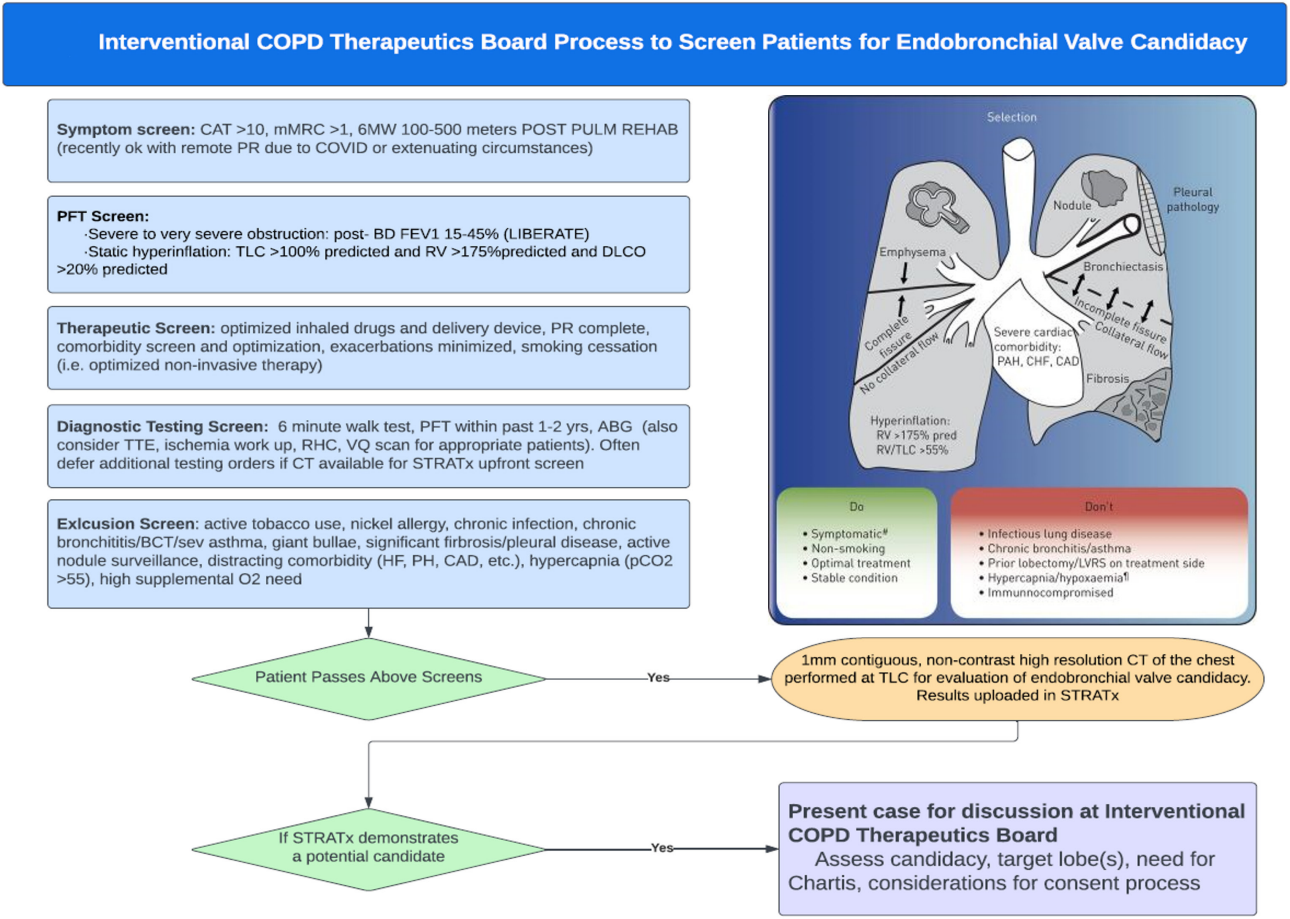
UCD interventional COPD therapeutics board process to screen patients for endobronchial valve candidacy.

This retrospective analysis was approved by the University of California, Davis Institutional Review Board (IRB Reference: 2246413‐1) and was exempt from requiring ethics approval. This is a retrospective analysis conducted at the University of California Davis Medical Center. We adopted a staged approach at our institution from 9/2022 to 9/2024 for all patients who were eligible for BLVR after evaluation. Patients were evaluated with pulmonary function tests (PFTs), chest CT, pulmonary rehabilitation, and optimisation of inhaler therapy. All patients met anatomical and PFT selection criteria, and a shared decision‐making process was used to discuss the risks and benefits of BLVR (see Figure 1) [7].

Data on patient demographics, procedural details, and complications were extracted from our electronic medical record system. This retrospective analysis of patients at UC Davis is the largest in existence for patients who have received staged BLVR in the US. As such, we report our retrospective analysis on this cohort to assess complications and patient outcomes. The following sections describe two illustrative cases from our experience.

### 2.2 Case #1

A 77‐year‐old male with highly symptomatic COPD and emphysema was referred for evaluation for BLVR. After comprehensive evaluation and medical optimisation, he underwent BLVR of the left upper lobe in a planned, two‐staged approach. In the first stage, one Zephyr endobronchial valve was placed in the LUL ([LB1 + 2, LB3]) after confirming the absence of collateral ventilation. The procedure was well tolerated, and the patient was discharged home the same day. Fourteen days later, the patient underwent a second stage to place the remaining valve in the LUL ([LB4 + LB5]).

**FIGURE 2:**
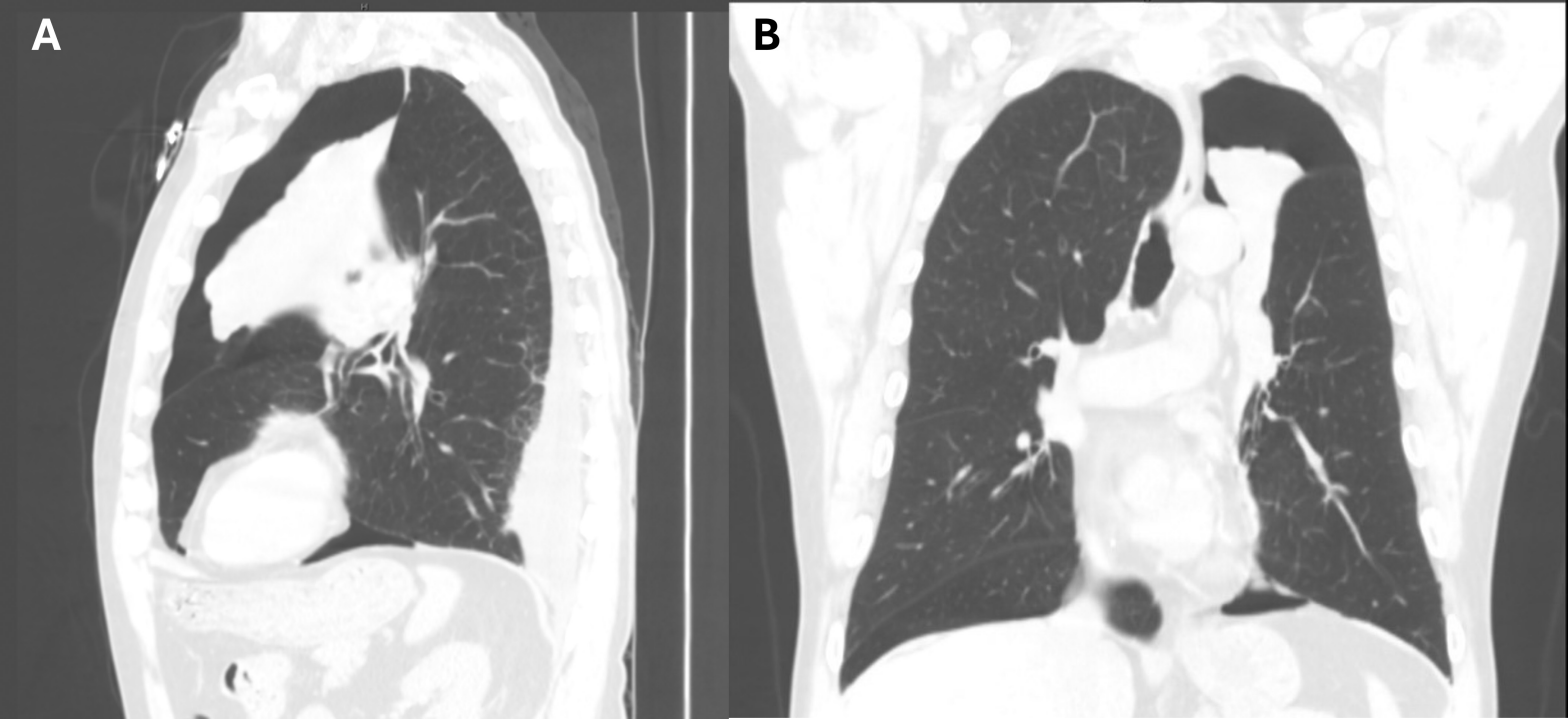
(A) Sagittal and (B) coronal CT images of patient #1 post BLVR showed complete left upper lobe atelectasis and left pneumothorax.

On hospital day three following the second stage, he developed acute dyspnea and chest pain and was found to have a moderate left pneumothorax (Figure 2). He was hemodynamically and respiratory stable, and a chest tube was deferred. The pneumothorax remained stable over the next 4 days without additional intervention, and he was discharged home on hospital day seven with strict return precautions. One week post‐discharge, the pneumothorax had completely resolved. At the one‐month follow‐up visit, the COPD Assessment Test (CAT) score improved from 26 to 19.

### 2.3 Case #2

A 62‐year‐old male with highly symptomatic COPD and emphysema was referred for evaluation for BLVR. After comprehensive evaluation and medical optimisation, the patient initially underwent a single‐stage BLVR of the LUL, with six Zephyr endobronchial valves placed (2 for [LB1 + 2], 3 for [LB3], and 1 for [LB4 + 5]).

**FIGURE 3:**
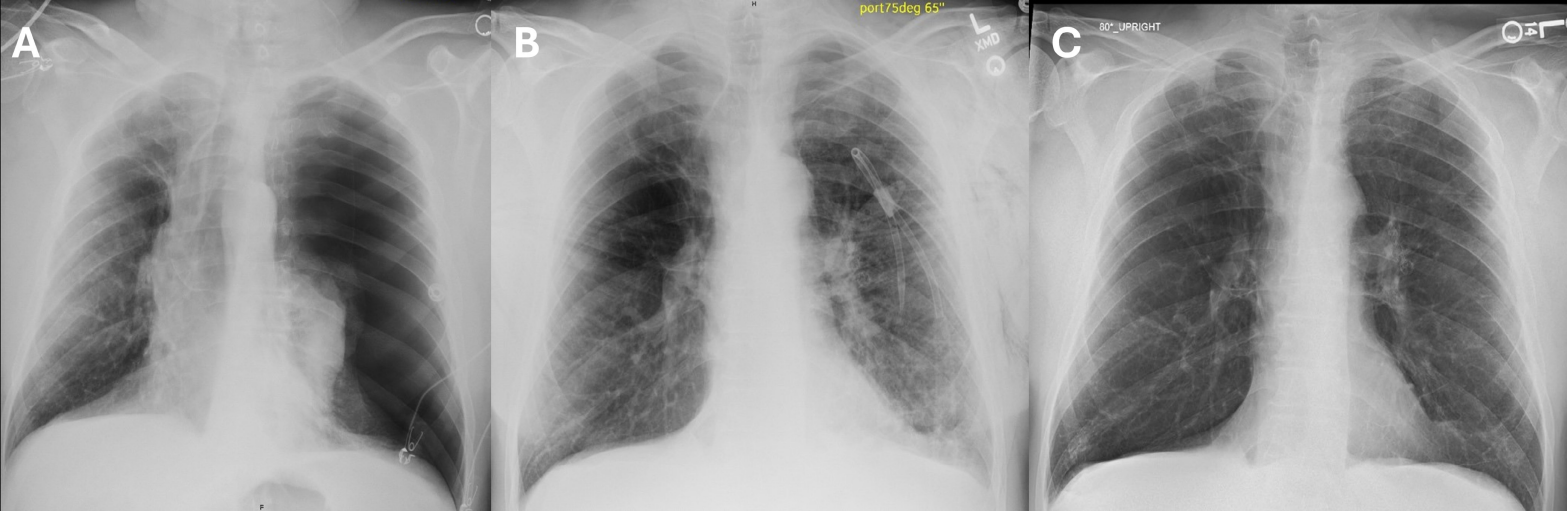
Case #2 patient radiographs. (A) Large left sided pneumothorax post single‐stage BLVR. (B) Persistent air leak after two valves removed, left tube thoracostomy allowed for lung expansion. (C) No pneumothorax after staged BLVR, tube thoracostomy removed.

Post‐procedurally, the patient developed a large left‐sided pneumothorax (Figure 3A) and was treated with a 14 French pigtail chest tube. Due to a persistent air leak, two endobronchial valves (lingula [LB4 + 5] and one of [LB3]) were removed on hospital day five. However, the air leak persisted over the next 2 days despite the patient's overall well‐being and ability to ambulate. Interval chest CT demonstrated partial re‐expansion of the LUL with a persistent left pneumothorax. Ultimately, a one‐way Heimlich valve was placed, and he was discharged home in stable condition on hospital day eight (Figure 3B).

Ten days post‐discharge, the left pneumothorax had resolved, and the patient returned to the bronchoscopy suite 18 days post‐discharge for replacement of the remaining two endobronchial valves in the LUL ([LB4 + 5] and [LB3]). The valve replacement was successful, and the patient had an uneventful two‐day hospital stay before discharge. No pneumothorax was observed, and the chest tube was removed prior to discharge (Figure 3C).

### 2.4 Results

We present our experience with 17 patients who underwent a staged BLVR procedure. Patient demographics and clinical characteristics are shown in Table 1. Sixteen patients were former smokers with an average of 40 pack‐years, and 94% of our patients were in GOLD stage III and IV based on FEV1 measurements. Overall, our patient population demonstrated severe obstruction (average FEV1: 0.92 L, 33% predicted), hyperinflation (average TLC: 7.66 L, 125% predicted), and air‐trapping (average RV: 5.23 L, 223% predicted), making them comparable in severity to those enrolled in the LIBERATE trial [2]. Similarly, patients exhibited elevated CAT and mMRC scores, consistent with prior major clinical trials. All patients were confirmed to be collateral ventilation negative with a Chartis System balloon occlusion assessment during the initial bronchoscopy, and mean target lobe fissure completeness was 94%.

**TABLE 1:** Baseline demographics and clinical characteristics.

Variables	Staged BLVR (*n* = 17)
Age, yr	71.41 ± 5.22
Sex, *n* (%)	
Male	9 (53)
Female	8 (47)
BMI, kg/m^2^	24.82 ± 4.18
Race, *n* (%)	
White	15 (88)
Black/African American	1 (6)
Other	1 (6)
Smoking pack‐years	40.4 ± 23.57
GOLD stage, *n* (%)	
Stage II	1 (6)
Stage III	9 (53)
Stage IV	7 (41)
Emphysema score of the target lobe at −910 HU^a^	65.35 ± 8.4
Target lobe fissure completeness, %	94.59 ± 5.17
Pre BLVR Post‐BD FEV_1_, L	0.92 ± 0.31
Pre BLVR Post‐BD FEV_1_, % predicted	32.88 ± 9.26
Pre BLVR Post‐BD FVC, L	2.51 ± 0.9
Pre BLVR Post‐BD FVC, % predicted	67.23 ± 19.19
DLCO, ml CO/min/mmHg	10.41 ± 5.69
DLCO, % predicted	37.94 ± 20.32
RV, L	5.23 ± 0.99
RV, % predicted	223.59 ± 33.56
TLC, L	7.66 ± 1.43
TLC, % predicted	125.59 ± 13.44
CAT score^b^	21.94 ± 6.38
mMRC score^c^	2.41 ± 1.00
6‐min‐walk‐distance, m	253 ± 102.03

*Note:* Values are means±SD.

Abbreviations: BLVR = bronchoscopic lung volume reduction; BMI = body mass index; CAT = COPD Assessment Test; GOLD = Global Initiative for Chronic Obstructive Lung Disease; HU = Hounsfield units; mMRC = modified Medical Research Council Dyspnea Scale; post‐BD = post‐bronchodilator.

^a^ Emphysema destruction score was assessed as the percentage of voxels of less than 2910 HU on computed tomography.

^b^ CAT scores range from 0 to 40, with higher score indicate more severe symptoms.

^c^ mMRC Dyspnea Scale ranges from 0 to 4, with higher scores indicating more severe dyspnea.

Table 2 details the procedural variables and outcomes. The majority (76%) of treated lobes were located in the left lung, with a distribution between the left upper lobe (35%) and left lower lobe (41%). A median of 3 total valves were placed but ranged between a minimum of 2 valves to a maximum of 6 valves were placed for each patient. Pneumothorax occurrence within 45 days was 11.8% and between 45 days and 12 months post procedure was 6%. Notably, no pneumothoraxes occurred between the first and second stages, and none of the pneumothorax events within the first 45 days required drainage. The median interval between stages was 21 days. Additional safety data revealed that the rates of COPD exacerbations and pneumonias were 12% and 6%, respectively, within 45 days, increasing to 29% and 12% between 45 days and 12 months. The mortality rate was 6% in both time periods.

**TABLE 2:** Staged BLVR characteristics and outcomes.

Variables	Staged BLVR (*n* = 17)
Days between stages, median (IQR)	21 (14–25)
Radiographic lobar collapse or diaphragm shift, *n* (%)	14 (82.3)
Number of valves placed, median (min–max)	3 (2–6)
Targeted lobe distribution	
RUL, *n* (%)	2 (12%)
RLL, *n* (%)	2 (12%)
LUL, *n* (%)	6 (35%)
LLL, *n* (%)	7 (41%)
Pneumothorax less than 45 days, *n* (%)	2 (12%)
Pneumothorax between 45 days and 12 months, *n* (%)	1 (6%)
Pneumothorax less than 45 days with need for drainage, *n* (%)	0
Pneumothorax between stage 1 and stage 2, *n* (% of all Pneumothorax)	0
Pneumothorax after stage 2, *n* (% of all Pneumothorax)	3 (100%)
COPD exacerbation less than 45 days, *n* (%)	2 (12%)
COPD exacerbation between 45 days and 12 months, *n* (%)	5 (29%)
Pneumonia less than 45 days, *n* (%)	1 (6%)
Pneumonia between 45 days and 12 months, *n* (%)	2 (12%)
Death less than 45 days, *n* (%)	1 (6%)
Death between 45 days and 12 months, *n* (%)	1 (6%)

*Note:* Time range is counted after stage 1 of BLVR.

Abbreviations: BLVR = bronchoscopic lung volume reduction; IQR = interquartile range; LLL = left lower lobe; LUL = left upper lobe; RLL = right lower lobe; RUL = right upper lobe.

## 3 Discussion

Post‐BLVR pneumothorax is thought to be caused by injury of the visceral pleura during the expansion of the untreated ipsilateral lobe [8, 9]. A staged valve placement is hypothesised to decrease the rate of atelectasis in the treated lung, perhaps allowing more time for adjacent lobe expansion, less alveolar tearing, and thus a lower rate of pneumothorax.

In our study, the rate of pneumothorax within 45 days was 11.8%, similar to that published by Egenod et al. (12.5%) and much lower than the accepted standard risk of pneumothorax (26.6%) based on the LIBERATE trial [2, 6]. Interestingly, none of the patients who developed pneumothorax within 45 days (2 out of 17) in our cohort required a chest tube drainage, which was different from the previously reported staged BLVR cohort, in which 100% of the pneumothorax were treated with chest tube drainage [6].

In our cohort, no pneumothorax occurred after the first stage of BLVR. Prior studies have similarly reported that most pneumothoraxes occur following the second stage of a staged approach. Furthermore, evidence suggests that significant target lobe volume reduction and improvement in PFTs can be achieved after the first stage alone [5]. This finding raises the possibility that, for some high‐risk patients, partial BLVR after the first stage might offer a favourable risk–benefit ratio, potentially obviating the need for a second procedure altogether.

The rate of COPD exacerbation in the short term (< 45 days) and long term (between 45 days and 12 months) groups was similar to the rates reported in the LIBERATE trial [12.5% vs. 11% and 31% vs. 35%]. However, there was a slightly higher pneumonia rate reported in our cohort when compared to the LIBERATE trial both in the short term (< 45 days) and long term (between 45 days and 12 months) groups [5.8% vs. 2.4% and 11.8% vs. 5.6%]. Other negative aspects of the staged approach include increased costs, more frequent hospital visits, greater utilisation of healthcare resources, increased exposure to anaesthesia, and a longer overall time to complete valve placement. These factors must be carefully weighed against the potential benefits of reduced pneumothorax rates, which may translate into shorter hospital stays and less resource utilisation for managing complications. From the patient's perspective, a small survey study indicated that 87% of patients would be willing to undergo a staged approach if it reduced the risk of pneumothorax [10]. Patients also tended to place less importance on factors such as cost, travel distance, and length of hospital stay, which further supports the acceptability of a staged BLVR strategy if its benefits are confirmed in larger studies.

Overall, these are promising results but there remains clinical equipoise, and a staged BLVR procedure is still not a common practice currently.

## Author Contributions

C.L. conceived study design, data collection and curation, data analysis, wrote manuscript. B.T.K. conceived study design, manuscript revision. M.S. conceived study design, manuscript revision. C.P. conceived study design, manuscript revision. K.Y.Y. conceived study design, manuscript revision. J.J.K. conceived study design, supervised data collection and curation, supervised data analysis, manuscript revision.

## Ethics Statement

The authors declare that they have obtained appropriate authorisation to access and publish the patients' data from: Institution review board or Ethics committee name: University of California, Davis Institutional Review Board. Authorisation number: (IRB Reference: 2246413–1).

## Consent

The authors declare that written informed consent was obtained for the publication of this manuscript and accompanying images using the form provided by the Journal for the patients described in cases 1 and 2.

## Conflicts of Interest

The authors declare no conflicts of interest.

## Data Availability

The data that support the findings of this study are available from the corresponding author upon reasonable request.
